# Supplementary material for: Two Facile Aniline-Based Hypercrosslinked Polymer Adsorbents for Highly Efficient Iodine Capture and Removal
Source: Int J Mol Sci. 2022 Dec 26;24(1):370. doi: 10.3390/ijms24010370 (PMC9820307; doi:10.3390/ijms24010370)
Supplement: Supplementary file 1 [file ijms-24-00370-s001.zip › ijms-2092887-supplementary.pdf]

Electronic Supplementary Information

Two Facile Aniline-Based Hypercrosslinked Polymer Adsorbents for Highly Efficient Iodine Capture and Removal

Biying Liu, Chaochao Mao, Zian Zhou, Qiannan Wang, Xiong Zhou, Zhijie Liao, Ran Deng, Defei Liu, Jingzi Beiyuan, Daofei Lv, Jiesen Li, Liyun Huang, * Xin Chen, Wenbing Yuan*

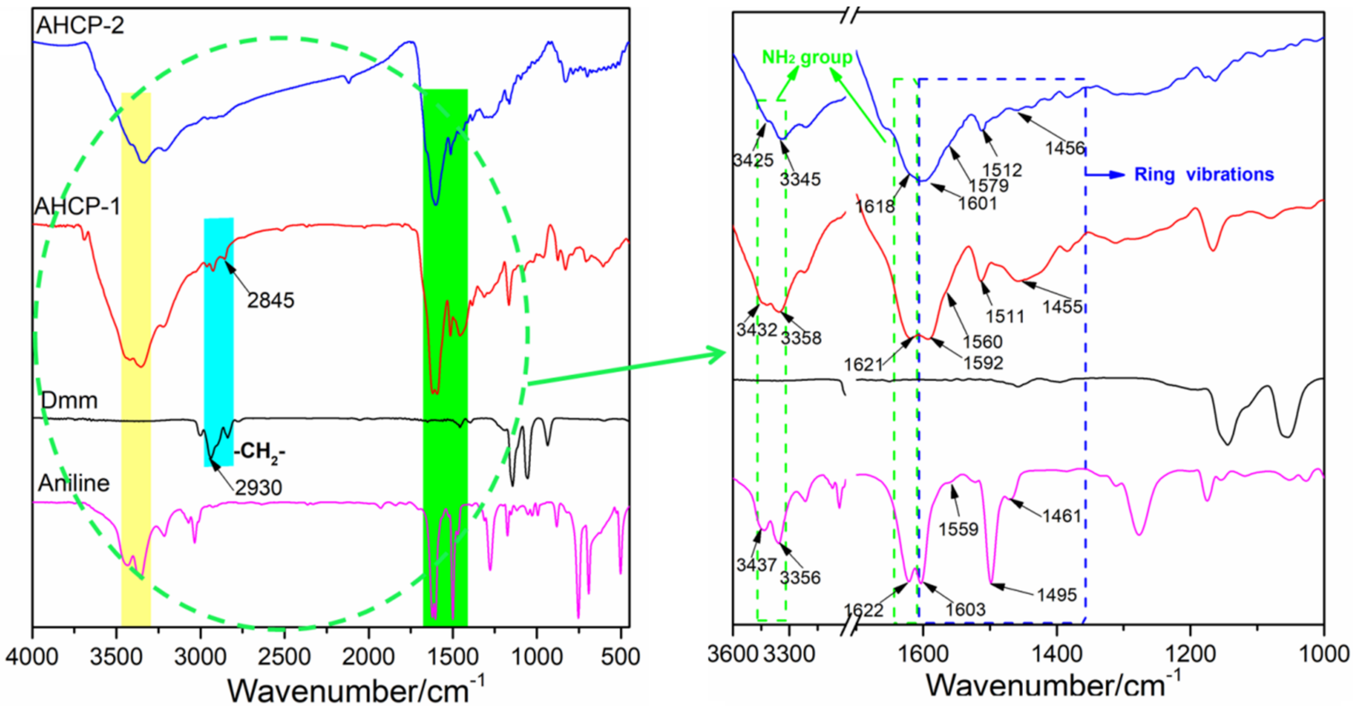

Fig. S1. FT-IR spectra of Aniline(purple), dimethoxymethane (Dmm) (black), AHCP-1(red), and AHCP-2(blue).

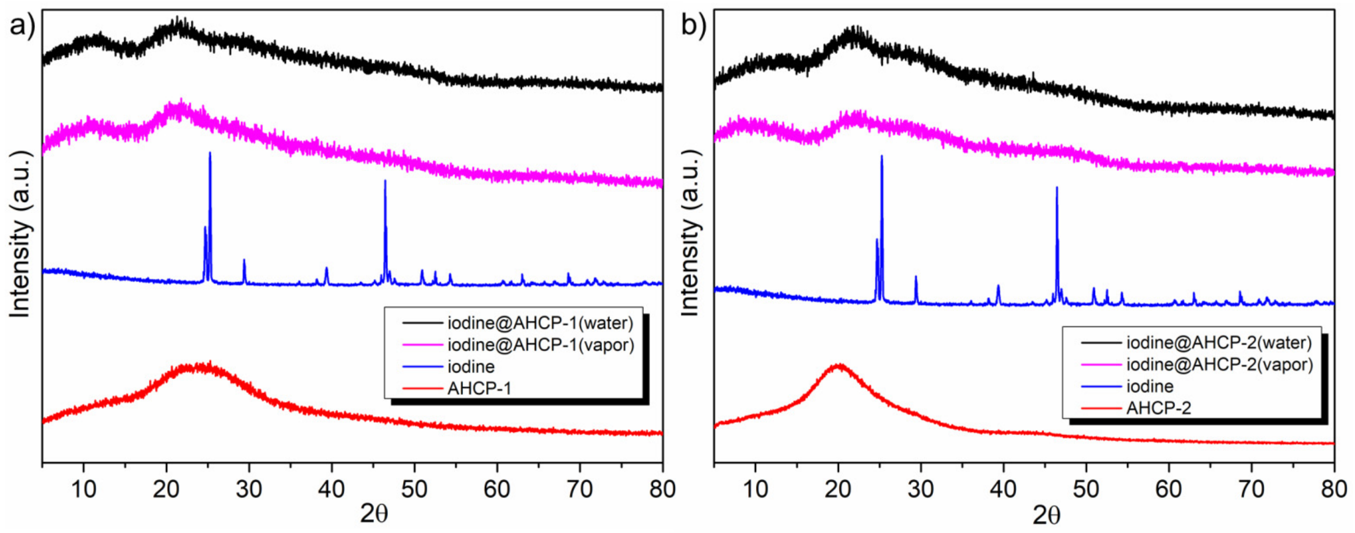

Fig. S2. Comparison of PXRD patterns of (a) AHCP-1 and (b) AHCP-2 before and after iodine adsorption.

Table S1. Iodine adsorption capacity of reported amorphous adsorbents in iodine aqueous solution

No.	Adsorbent	BET(m^2/g)	Iodine adsorption capacity (wt%)	Reference (DOI)
**1**	**AHCP-1**	**14.4**	**250**	**This work**
**2**	Fe_3O_4@PPy	30	162.7	10.1016j.jhazmat.2017.10.065
**3**	**AHCP-2**	**75.2**	**160**	**This work**
**4**	BC@Dopa-ZIF	1453.3	131	10.1021/acs.cgd.7b01360
**5**	Activated Bamboo	-	118	10.1016/S0008-6223(00)00230-X
**6**	Activated Salsola vermiculata plant	-	117.8	10.1016/j.w atres.2014.10.021
**7**	Activated Coconut shell	-	117	10.1016/S0008-6223(00)00230-X
**8**	bituminous coal-based granular (GAC-B)	960	101.6	10.1016S0008-6223(99)00180-3
**9**	Activated Japanese cedar	590	95	10.1016/S0008-6223(00)00230-X
**10**	activated carbon the granular carbon made of coconut shells (GAC-C)	940	94	10.1016S0008-6223(99)00180-3
**11**	THPS-C	3125	92.6	10.1002/admi.201900249
**12**	The activated carbon fabric made from PAN(ACF)	970	88.9	10.1016S0008-6223(99)00180-3
**13**	Activated Vinylidene film	714	85	10.1016/S0008-6223(00)00230-X
**14**	Activated Evergreen oak	-	85	10.1016/S0008-6223(00)00230-X
**15**	Merck activated carbon	-	20	10.1016/j.w atres.2014.10.021
**16**	n-CF@OCDs	-	19.01	10.1016/j.jhazmat.2019.01.037
**17**	im-SCMNPs	-	14.1	10.1007/BF03353693
**18**	n-CF	-	2.81	10.1016/j.jhazmat.2019.01.037
**19**	PHCP@PES	13.75	18	10.1016/j.jhazmat.2022.128859
**20**	CTF-DPA	943	66.7	10.1016/j.jece.2022.107805
**21**	P-TzTz	137	49.09	10.1016/j.micromeso.2020.110161

Table S2. Iodine adsorption capacity of reported amorphous adsorbents in iodine vapor

No.	Adsorbent	BET (m^2/g)	Iodine adsorption capacity (wt%)	Reference (DOI)
**1**	**AHCP-1**	**14.4**	**536**	**This work**
**2**	**AHCP-2**	**75.2**	**596**	**This work**
**3**	TTPPA	512.39	490	10.1039/c7ta08251g
**4**	TTPB	222	443	10.1039/C7TA00590C
**5**	CTF-DPA	943	512	10.1016/j.jece.2022.107805
**6**	HCP	584	460	10.1016/j.seppur. 2018.09.041
**7**	BPPOC	-	564	10.1021/jacs.2c03959
**8**	CMP-4	9.5	208	10.1002/ange.202015162
**9**	PHF-1-Ct	690	405	10.1039/C8CC07529H
**10**	C-poly-15	96.16	574	10.1039/D1SC05361B
**11**	PHCP@PES	13.75	77	10.1016/j.jhazmat.2022.128859
**12**	P-TzTz	137	326	10.1016/j.micromeso.2020.110161
**13**	CSU-CPOPs-1	1032	494	10.1021/acsami.9b07679
**14**	FcTz-POP	-	264	10.1016/j.micromeso.2021.110929
**15**	C-poly-15	96.16	574	10.1039/d1sc05361b
**16**	PZ-HCP-1:4	209	333	10.1016/j.micromeso.2022.112157
**17**	ImCMP-1	356.9	236	10.1016/j.micromeso.2022.111871
**18**	TTDAB	1.643	313	10.1039/c7ta08251g
**19**	Tm-MTDAB	2.778	304	10.1039/c7ta08251g
**20**	CalPOF-1	303	477	10.1021/acssuschemeng.8b05203
**21**	CalPOF-2	154	406	10.1021/acssuschemeng.8b05203
**22**	CalPOF-3	91	353	10.1021/acssuschemeng.8b05203
**23**	NDB-H	116.93	443	10.1002/asia.201800698
**24**	NDB-S	56.45	425	10.1002/asia.201800698
**25**	ADB-HS	148.2	345	10.1002/asia.201800698
**26**	ADB-S	41.53	342	10.1002/asia.201800698
**27**	POP-1	12	357	10.1016/j.jhazmat.2017.05.041
**28**	POP-2	41	382	10.1016/j.jhazmat.2017.05.041
**29**	HCMP-1	430	159	10.1021/acs.macromol.6b00901
**30**	HCMP-2	153	281	10.1021/acs.macromol.6b00901
**31**	HCMP-3	82	316	10.1021/acs.macromol.6b00901

**32**	HCMP-4	28	222	10.1021/acs.macromol.6b00901
**33**	CalP4_Li	445	312	10.1021/acs.chemmater.7b03449
**34**	TBIM	8.12	943	10.1039/c9ta11982e
**35**	AzoPPN	400	290	10.1002/chem.201602337
**36**	Azo-Trip	510.4	238	10.1039/C5PY01671A
**37**	CMP-E1	2150	215	10.1002/anie.201503362
**38**	PAF-21	1520	152	10.1002/anie.201503362
**39**	PAF-23	82	271	10.1002/anie.201503362
**40**	PAF-24	136	276	10.1002/anie.201503362
**41**	PAF-25	262	260	10.1002/anie.201503362
**42**	NAPOP-1	657	206	10.1002/pola.28028
**43**	NAPOP-2	458	205	10.1002/pola.28028
**44**	NAPOP-3	702	241	10.1002/pola.28028
**45**	NAPOP-4	626	265	10.1002/pola.28028
**46**	CMPN-1	230	97	10.1039/C4TA04235B
**47**	CMPN-2	339	110	10.1039/C4TA04235B
**48**	CMPN-3	1368	208	10.1039/C4TA04235B
**49**	NiP-CMP	2630	202	10.1039/c4cc01783h
**50**	PAF-1	5600	186	10.1039/C4TA00049H,<br>10.1002/anie.200904637
**51**	JUC-Z2	2081	144	10.1039/C4TA00049H,<br>10.1039C1JM12545A
**52**	CMOP-1	431	160	10.3390/molecules23071732
**53**	CMOP-2	406	93	10.3390/molecules23071732
**54**	NTP	1067	180	10.1021/acsmacrolett.6b00567
**55**	AK-1	1634	237	10.1016j.jhazmat.2016.09.015
**56**	AK-2	2751	262	10.1016j.jhazmat.2016.09.015
**57**	AK-3	2016	253	10.1016j.jhazmat.2016.09.015
**58**	PAN1	618	133	10.1002app.46106
**59**	PAN2	542	245	10.1002app.46106
**60**	PAN3	194	281	10.1002app.46106
**61**	PAN4	522	237	10.1002app.46106
**62**	PAN5	439	189	10.1002app.46106
**63**	Cg-5P	840	8.7	10.1039c1ra00351h
**64**	Cg-5C	1290	23.6	10.1039c1ra00351h
**65**	SCMP-1	418	188	10.1021acsami.6b06569

**66**	SCMP-2	855	220	10.1021acsami.6b06569
**67**	SCMP-II	119.76	354	10.1039/C6CC05188J
**68**	BQCMP-1	422	151	10.1002/mame.201600138
**69**	BQCMP-2	123	161	10.1002/mame.201600138
**70**	FCMP-600@1	551	108	10.1038/s41598-017-14598-0
**71**	FCMP-600@2	636	141	10.1038/s41598-017-14598-0
**72**	FCMP-600@3	629	90	10.1038/s41598-017-14598-0
**73**	FCMP-600@4	88	111	10.1038/s41598-017-14598-0
**74**	NCMP1	58	215	10.1021/acsami.7b09553
**75**	NCMP2	280	186	10.1021/acsami.7b09553
**76**	NCMP3	485	161	10.1021/acsami.7b09553
**77**	NOP-54	1178	202	10.1016/j.cej.2017.10.133
**78**	NiMoS chalcogels	490	225	10.1021/acs.chemmater.5b00413
**79**	MelPOP-2	50.5	262	10.1039/C8CC04242J
**80**	PSIF-1a	320	485	10.1021/acsami.8b03023

Sorption kinetics

Pseudo first order kinetic model

The linear form of pseudo first order kinetic model is expressed by the following equation.

$$\ln(q_e - q_t) = \ln q_e - k_1 t$$

Where q_e and q_t are the amount of iodine adsorbed at time t and equilibrium (g/g), k_1 is the pseudo-first-order rate constant of adsorption process(min^{-1})

Pseudo second order kinetic model

The linear form of pseudo second order kinetic model is expressed by the following equation.

$$\frac{t}{q_t} = \frac{1}{k_2 q_e^2} + \frac{1}{q_e} t$$

Where q_e and q_t are the amount of iodine adsorbed at time t and equilibrium (mg/g), k_2 is the pseudo-first-order rate constant of adsorption process (g/mg min)

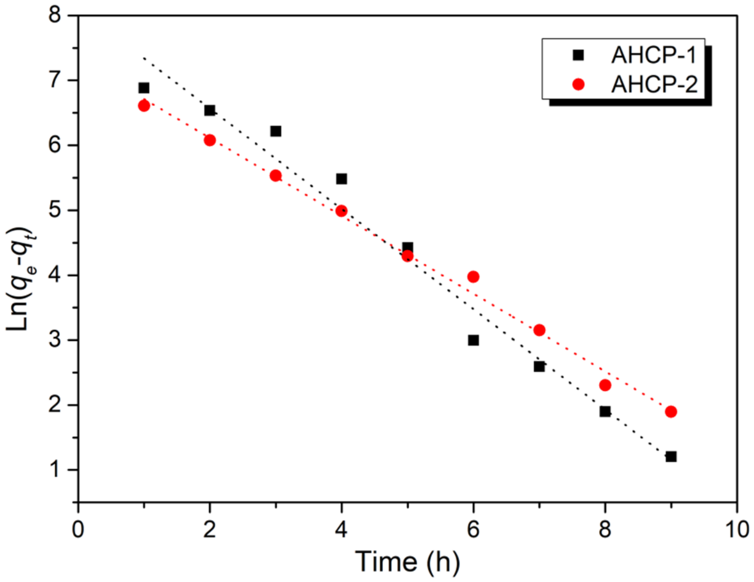

Fig. S3. Plots of pseudo-first order kinetics for the adsorption of I_2 aqueous solution on AHCP-1 and AHCP-2.

Table S3. Parameters of the pseudo first order and pseudo second order kinetic models extracted from experimental data for AHCP-1 and AHCP-2.

		**Intercept**	q_e **(g/g)**	k_1**(1/h)**	R^2
**pseudo-first order**	AHCP-1	8.1118	3.33	0.7729	0.9762
	AHCP-2	7.3119	1.49	0.5994	0.9939
		Intercept	q_e (g/g)	K_2(g/mg·h)	R^2
**Pseudo second order**	AHCP-1	0.0003	2.33	0.0006	0.9972
	AHCP-2	0.0006	1.67	0.0005	0.9990

Sorption equilibrium

Langmuir isotherm

The equation of Langmuir is expressed by the following equation.

$$\frac{1}{q_e} = \frac{1}{q_m} + \frac{1}{k_L q_m C_e}$$

Freundlich isotherm

The equation of Freundlich is expressed by the following equation.

$$\ln q_e = \ln k_F + \frac{1}{\mathrm{n}} \ln C_e$$

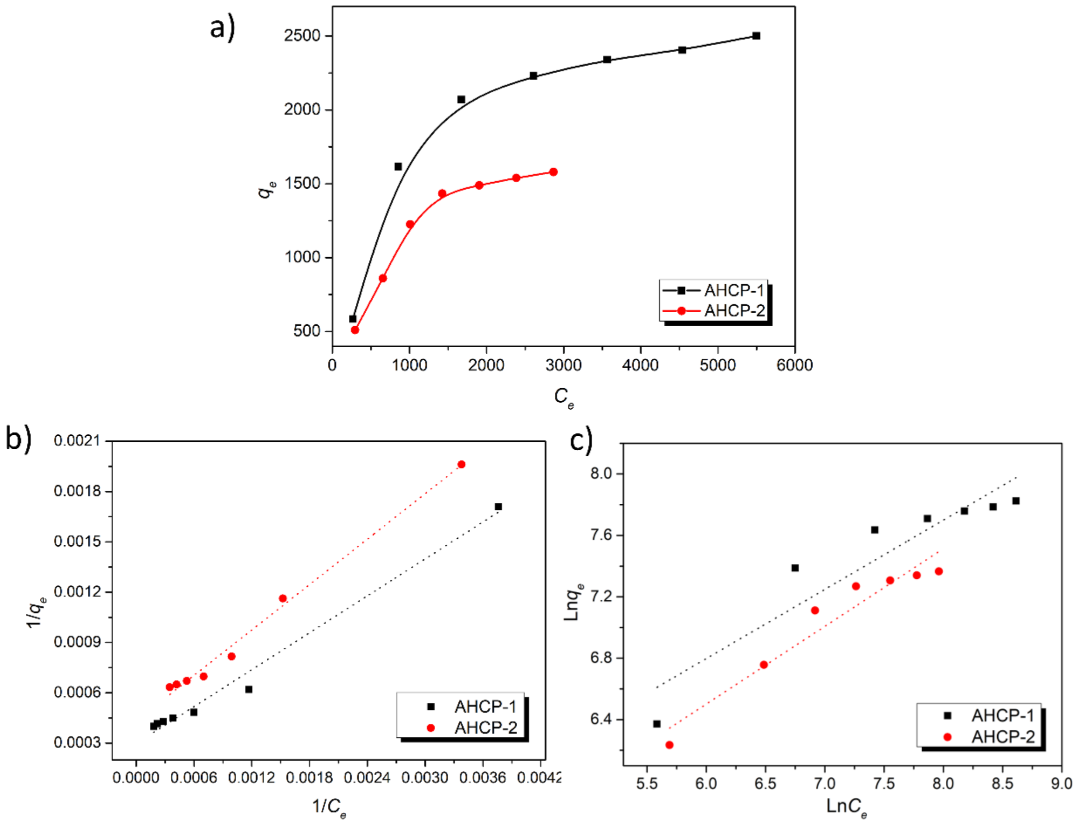

Fig. S4. (a) Adsorption Isotherm iodine onto AHCPs. (b) Plots of Langmuir Isotherms for the adsorption of I_2 aqueous solution on AHCP-1 and AHCP-2. (c) Plots of **Freundlich** Isotherms for the adsorption of I_2 aqueous solution on AHCP-1 and AHCP-2.

Table S4. Parameters of the different isotherm models extracted from experimental adsorption isotherms data for AHCP-1 and AHCP-2.

	Langmuir isotherm			Freundlich isotherm		
	Q_m(g/g)	k_L(1/mg)	R^2	k_F(mg/g)	n	R^2
AHCP-1	2.70	0.0011	0.9872	59.9074	2.2178	0.8766
AHCP-2	2.22	0.0010	0.9922	31.9317	1.9751	0.9330

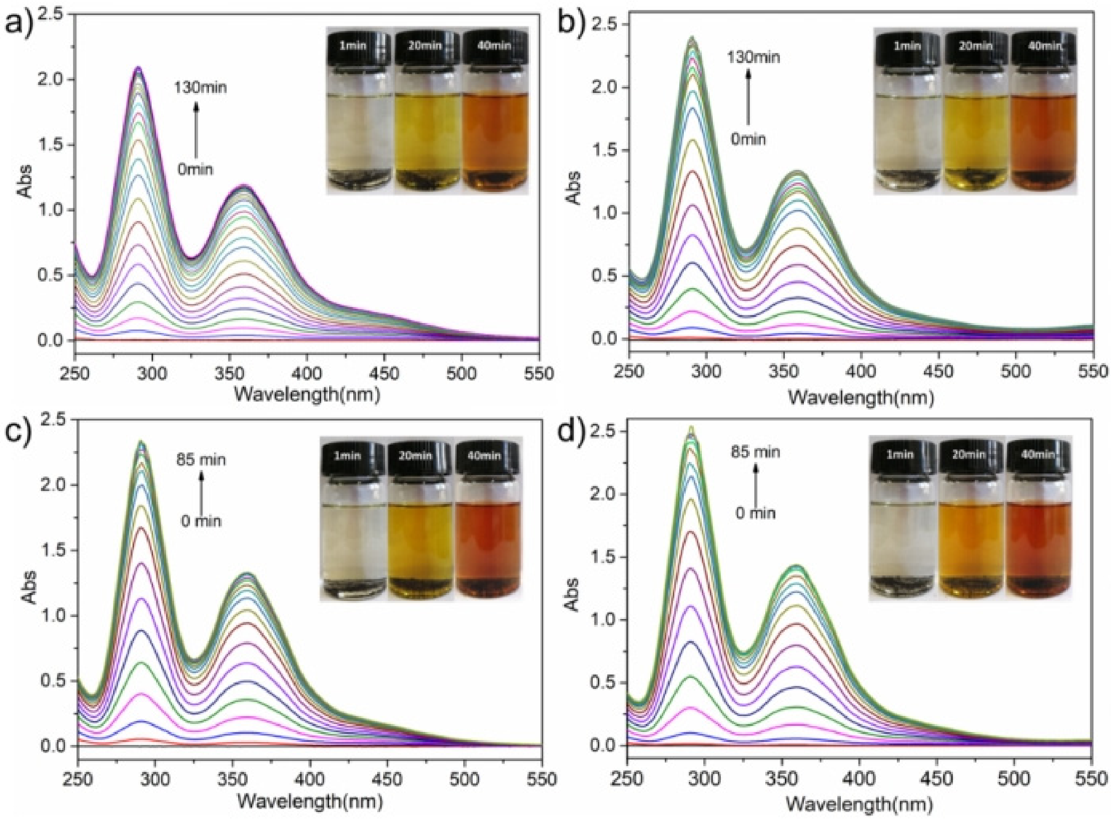

Fig. S5. Time-dependent UV−vis absorption of iodine@AHCPs in methanol. (a) iodine@AHCP-1(water) (10mg) in MeOH (45mL). (b) iodine@AHCP-2(water) (10mg) in MeOH (45mL). (c) iodine@AHCP-1(vapor) (10mg) in MeOH (80mL). (d) iodine@AHCP-2 (vapor) (10mg) in MeOH (80mL). Insets: photographs show color change of iodine enrichment at various times.

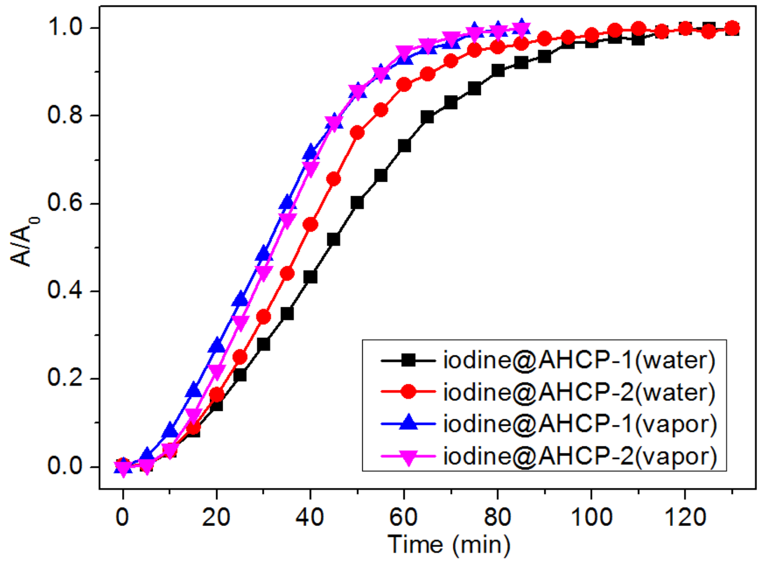

Fig. S6. Iodine desorption efficiencies at various times of iodine@AHCP-1(water), iodine@AHCP-2(water), iodine@AHCP-1(vapor), iodine@AHCP-2(vapor) monitored at 291nm.

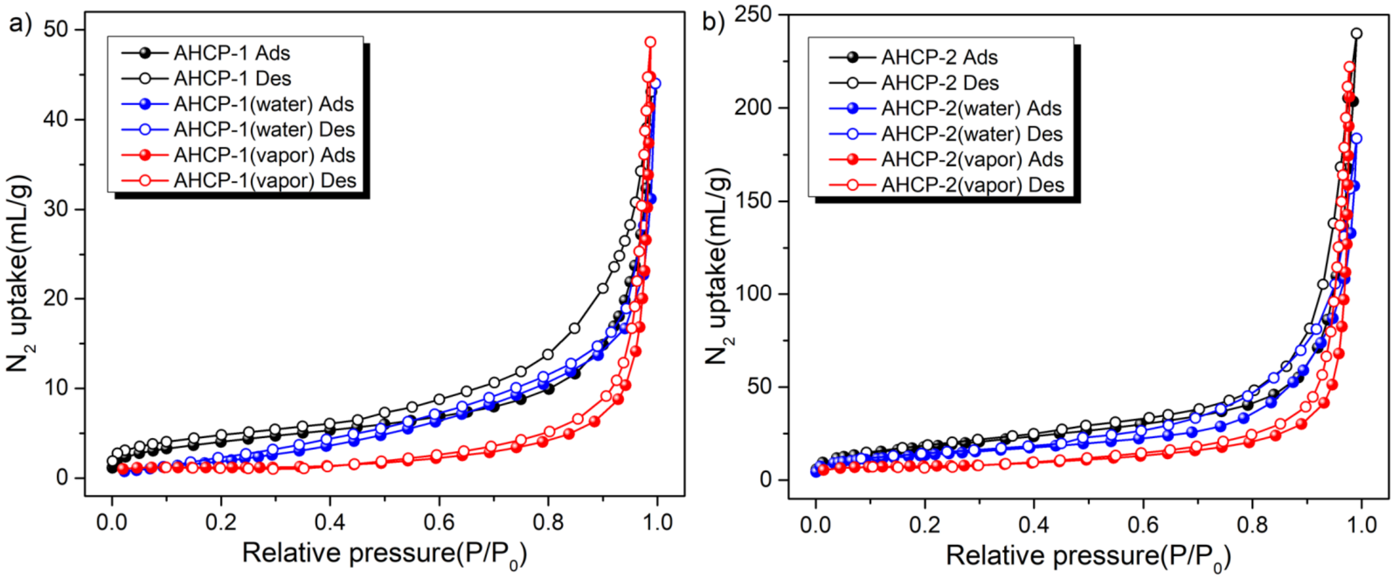

Fig. S7. BET curve after the first adsorption-desorption of iodine by AHCPs.

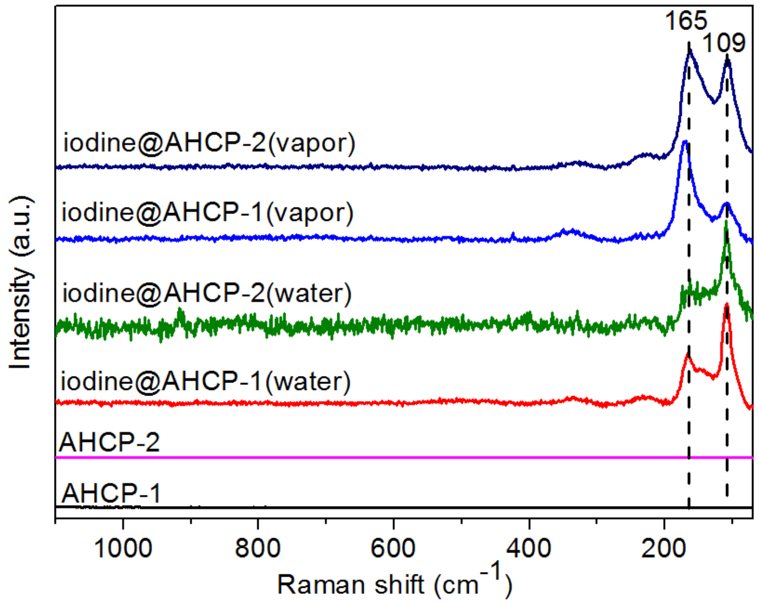

Fig. S8. Raman spectra of AHCPs, iodine@AHCPs(water) and iodine@AHCPs(vapor)

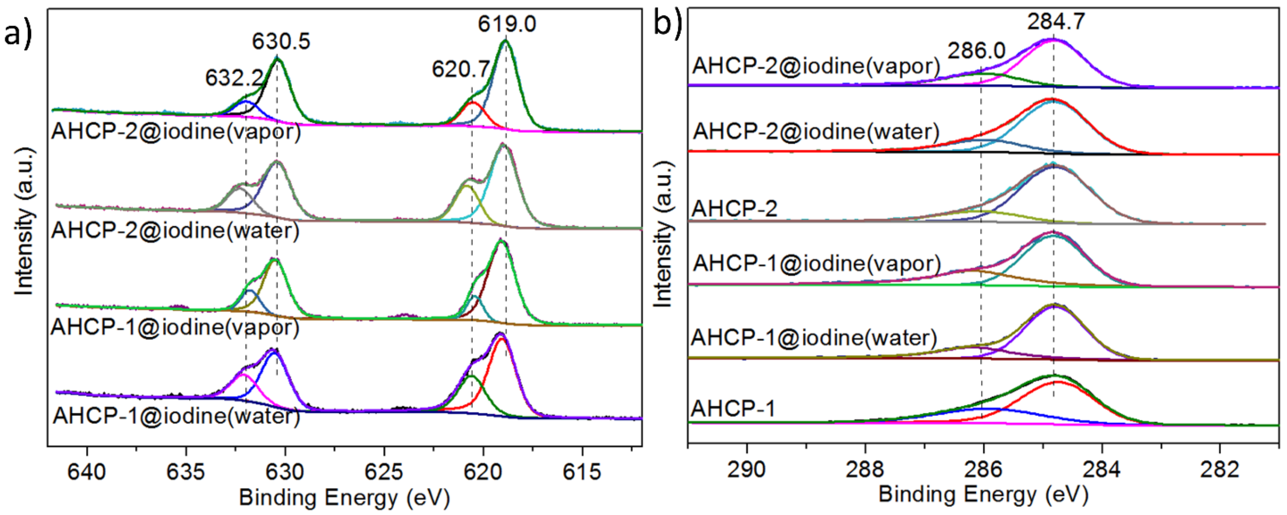

Fig. S9. XPS spectra of AHCPs, iodine@AHCPs(water) and iodine@AHCPs(vapor), (a) I3d; (b) C 1s,

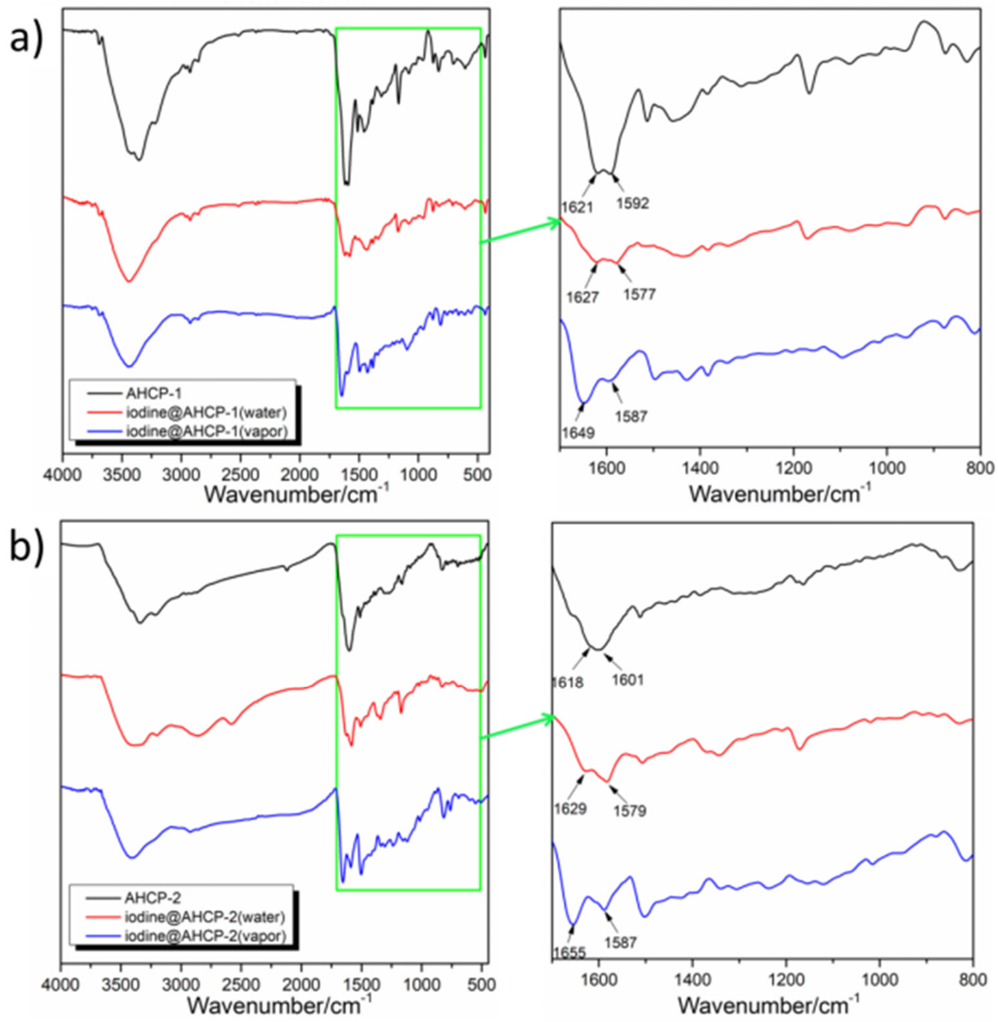

Fig. S10. (a) FT-IR spectra comparison of AHCP-1 before and after iodine adsorption; (b) FT-IR spectra comparison of AHCP-2 before and after iodine adsorption.
